# Decreased Hsp90 activity protects against TDP-43 neurotoxicity in a *C*. *elegans* model of amyotrophic lateral sclerosis

**DOI:** 10.1371/journal.pgen.1011518

**Published:** 2024-12-26

**Authors:** Laura Garcia-Toscano, Heather N. Currey, Joshua C. Hincks, Jade G. Stair, Nicolas J. Lehrbach, Nicole F. Liachko

**Affiliations:** 1 Geriatrics Research Education and Clinical Center, Veterans Affairs Puget Sound Health Care System, Seattle, Washington, United States of America; 2 Division of Gerontology and Geriatric Medicine, Department of Medicine, University of Washington, Seattle, Washington, United States of America; 3 Basic Sciences Division, Fred Hutch Cancer Center, Seattle, Washington, United States of America; Brown University, UNITED STATES OF AMERICA

## Abstract

Neuronal inclusions of hyperphosphorylated TDP-43 are hallmarks of disease for most patients with amyotrophic lateral sclerosis (ALS). Mutations in *TARDBP*, the gene coding for TDP-43, can cause some cases of familial inherited ALS (fALS), indicating dysfunction of TDP-43 drives disease. Aggregated, phosphorylated TDP-43 may contribute to disease phenotypes; alternatively, TDP-43 aggregation may be a protective cellular response sequestering toxic protein away from the rest of the cell. The heat shock responsive chaperone Hsp90 has been shown to interact with TDP-43 and stabilize its normal conformation; however, it is not known whether this interaction contributes to neurotoxicity *in vivo*. Using a *C*. *elegans* model of fALS mutant TDP-43 proteinopathy, we find that loss of function of HSP-90 protects against TDP-43 neurotoxicity and subsequent neurodegeneration in adult animals. This protection is accompanied by a decrease in both total and phosphorylated TDP-43 protein. We also find that *hsp-90* mutation or inhibition upregulates key stress responsive heat shock pathway gene expression, including *hsp-70* and *hsp-16*.*1*, and we demonstrate that normal levels of *hsp-16*.*1* are required for *hsp-90* mutation effects on TDP-43. We also observe that the neuroprotective effect due to HSP-90 dysfunction does not involve direct regulation of proteasome activity in *C*. *elegans*. Our data demonstrate for the first time that Hsp90 chaperone activity contributes to adverse outcomes in TDP-43 proteinopathies *in vivo* using a whole animal model of ALS.

## Author summary

Most patients with the neurodegenerative disease amyotrophic lateral sclerosis (ALS) develop aggregates of the protein TDP-43 within neurons in the brain and spinal cord. To study the process controlling these changes we have developed simple invertebrate models of ALS using *C*. *elegans* that express human TDP-43 in all neurons. These models accumulate TDP-43 and exhibit many features of human disease including motor neuron dysfunction and neurodegeneration. In this study we examine the role of the chaperone HSP-90, a protein that assists other proteins to form and maintain appropriate structures under normal or stress conditions. We show that genetic or small molecule inhibition of HSP-90 is able to reduce disease- modified TDP-43 and prevent some of the disease phenotypes. We also find that another gene, the small heat shock protein *hsp-16*.*1*, is required for this effect. These results identify a role for HSP-90 that may contribute to ALS pathogenesis, and examines cellular pathways by which this occurs.

## Introduction

Amyotrophic lateral sclerosis (ALS) is a highly disabling disease characterized by the progressive loss of upper cortical and lower spinal motor neurons. Loss of motor neurons is accompanied by muscle denervation, causing weakness, spasticity, and finally, paralysis, which can lead to fatal respiratory failure typically within 3–5 years after diagnosis [1]. Most patients with ALS have a sporadic form of the disease with no known genetic cause. However, there are currently more than 30 gene mutations thought to cause familial inherited forms of ALS [2].

Similar to other neurodegenerative proteinopathies, such as Alzheimer’s disease, Parkinson’s disease, and Huntington’s disease, ALS exhibits accumulation of neurotoxic misfolded proteins in disease affected regions of the central nervous system [3]. The vast majority of both sporadic and familial ALS-diagnosed cases (about 97%) present inclusions containing the protein TAR DNA-binding protein of 43 kDa (TDP-43) in degenerating neurons, thus constituting the main pathological hallmark of the disease. TDP-43 is an essential protein contributing to many aspects of RNA metabolism, including mRNA splicing regulation and RNA processing [4]. TDP-43 protein levels are tightly controlled through a negative feedback loop, in which TDP-43 recognizes its own RNA transcript and destabilizes it, thus reducing TDP-43 protein production [5]. Under physiological conditions, this protein resides primarily in the nucleus but can be transported into the cytoplasm, where it contributes to RNA stability and transport. ALS-associated TDP-43 accumulates as insoluble inclusions in neurons and other central nervous system cells, including microglia, astrocytes, and oligodendrocytes, thus contributing to neuronal dysfunction and interfering in myelin regeneration [6,7]. Within these inclusions, TDP-43 exhibits a variety of post-translational modifications, such as acetylation, ubiquitination, phosphorylation, disulfide bridge formation, and sumoylation [8]. Of these, phosphorylation is used diagnostically to identify TDP-43 pathology in disease. Accumulation of phosphorylated TDP-43 not only results in a loss of normal nuclear functions of TDP-43 but can disrupt its autoregulation and protein turnover mechanisms [5].

Neuronal health relies on maintenance of TDP-43 protein levels. In support of this, both autophagy and the ubiquitin-proteasome system (UPS) have been implicated in normal TDP-43 homeostasis and in disease [9–11]. While autophagy clears misfolded, insoluble or aggregated proteins from the cell, the UPS primarily controls degradation of unfolded polypeptides, which must be capable of entering into the proteasome. Soluble TDP-43 is preferentially degraded through the UPS, although the UPS can also clear disaggregated pathological TDP-43 [12, 13].

The Hsp90 family encompasses a heterogeneous group of phylogenetically highly conserved molecular chaperones acting as a key player in the regulation of degradation of misfolded proteins [14–17]. In humans, this family includes two cytosolic Hsp90 members (HSP90AA1 and HSP90AB1), one specifically located in the endoplasmic reticulum (ER) (HSP90B1), and one mitochondrial resident (TRAP1) [18]. HSP90AA1 and HSP90AB1 can be localized in the nucleus [18–20] as part of their chaperone role facilitating the transport of certain cargos, like steroid receptors [21]. In *C*. *elegans* there is only one cytosolic Hsp90 homolog, *hsp-90* (also called *daf-21*) [22,23], one ER resident (*enpl-1*), and one mitochondrial family member (*hsp-75*) [24,25].

Mammalian Hsp90 is widely expressed throughout the central nervous system and participates in a variety of cellular processes including cell signaling, cytoskeleton integrity maintenance, proteasome maintenance, and cell cycle [18,26]. Through its chaperone activities, it functions with a network of co-chaperones to regulate both *de novo* synthesized and incorrectly folded client protein folding. Hsp90 plays a crucial role in the quality control of proteins, including preventing protein misfolding or assisting in protein degradation in order to counteract protein aggregation [27,28]. Hsp90 is highly expressed, representing about 1% of the total cellular proteins in eukaryotes [29,30], and its activity is tightly regulated by its interaction with other chaperones and co-chaperones. Under stress conditions, Hsp90 works together with the chaperone Hsp70 to form an essential part of the cellular quality control system and one of the main lines of defense against misfolded proteins through a co-chaperone-dependent process, including the co-chaperones STI-1/HOP [16,31] and CHIP [16,31–33].

In *C*. *elegans*, *hsp-90* participates in a broad variety of processes. During development, it is expressed preferentially in germ cell progenitors, playing a role in reproduction. In addition to expression in cell types including neurons, muscle, and the intestine, HSP-90 is present in germ line cells in adulthood, corroborating its role controlling *C*. *elegans* reproduction [34]. As in mammals, it participates in cell division, cell proliferation, and muscle proteostasis [35–37]. Moreover, HSP-90 can function as a life span regulator by controlling DAF-16 isoform A nuclear import [38]. HSP-90 is also involved in sensory cilia, where it contributes to the normal chemosensory signal transduction, which is essential for survival [39, 40]. HSP-90 also has important roles in protein quality control in *C*. *elegans*. Under stress conditions, perinuclear HSP-90 protects newly synthesized proteins from denaturation or from being degraded by the cell stress response in somatic cells [35]; moreover, this protective role against chronic proteotoxic stress functions as an integrated organismal response in a cell non-autonomous manner [41].

While molecular chaperones such as Hsp90 have been studied in ALS [31], a full understanding of their roles remains unclear. Evidence exists that molecular chaperones can function both as protectors against the toxicity associated with protein misfolding and as enhancers of disease that maintain the stability of the toxic form of TDP-43 [31]. For example, Hsp90 inhibition reduces TDP-43 levels in an autophagy-dependent mechanism in HeLa cell models [42]. In contrast, another model of TDP-43 proteinopathy generated in human neuroblastoma cells found the knockdown of both Hsp70 and Hsp90 chaperones leads to an accumulation of toxic TDP-43 species, potentially by blocking the degradation pathways in which these chaperones are involved [12]. However, consequences of Hsp90 inhibition have not been tested *in vivo*, in the context of an intact nervous system.

In this study, we explore the role of the *C*. *elegans hsp-90*/*daf-21* chaperone in an animal model of TDP-43 proteinopathy that expresses full length human TDP-43 containing ALS-causing mutations pan-neuronally. We found that disrupting HSP-90 functions genetically or with small molecule inhibitors is sufficient to protect against TDP-43 neuronal dysfunction and neurodegeneration.

## Results

### HSP-90 mutation or inhibition improves hTDP-43 driven motor dysfunction in C. elegans

Hsp90 is an essential gene, and complete loss of function of Hsp90 is not tolerated in eukaryotes [39,43,44]. A single cytoplasmic Hsp90 homolog exists in *C*. *elegans*, the gene *hsp-90* (previously named *daf-21* in *C*. *elegans*). Although complete loss of *hsp-90* is larval lethal, a point mutation within the ATP binding domain of *hsp-90*, *hsp-90(p673)*, mutates a conserved glutamic acid residue to lysine (E292K), changing the charge at this site from negative to strongly positive [39]. This mutation results in a temperature-sensitive alteration of HSP-90 function, whereby HSP-90 exhibits reduced ATPase activity but maintains its stability, chaperone functions, and interactions with co-factors [36]. When maintained at the permissive temperature (16°C) through larval development, *hsp-90(p673)* animals display incomplete penetrance of constitutive dauer formation (Daf-c), where a subset of animals enter into dauer, an alternate non-reproductive and highly stress resistant developmental state. However when grown at higher temperatures during development (25°C), *hsp-90(p673)* animals display close to 100% penetrance dauer formation [45].

**Fig 1:**
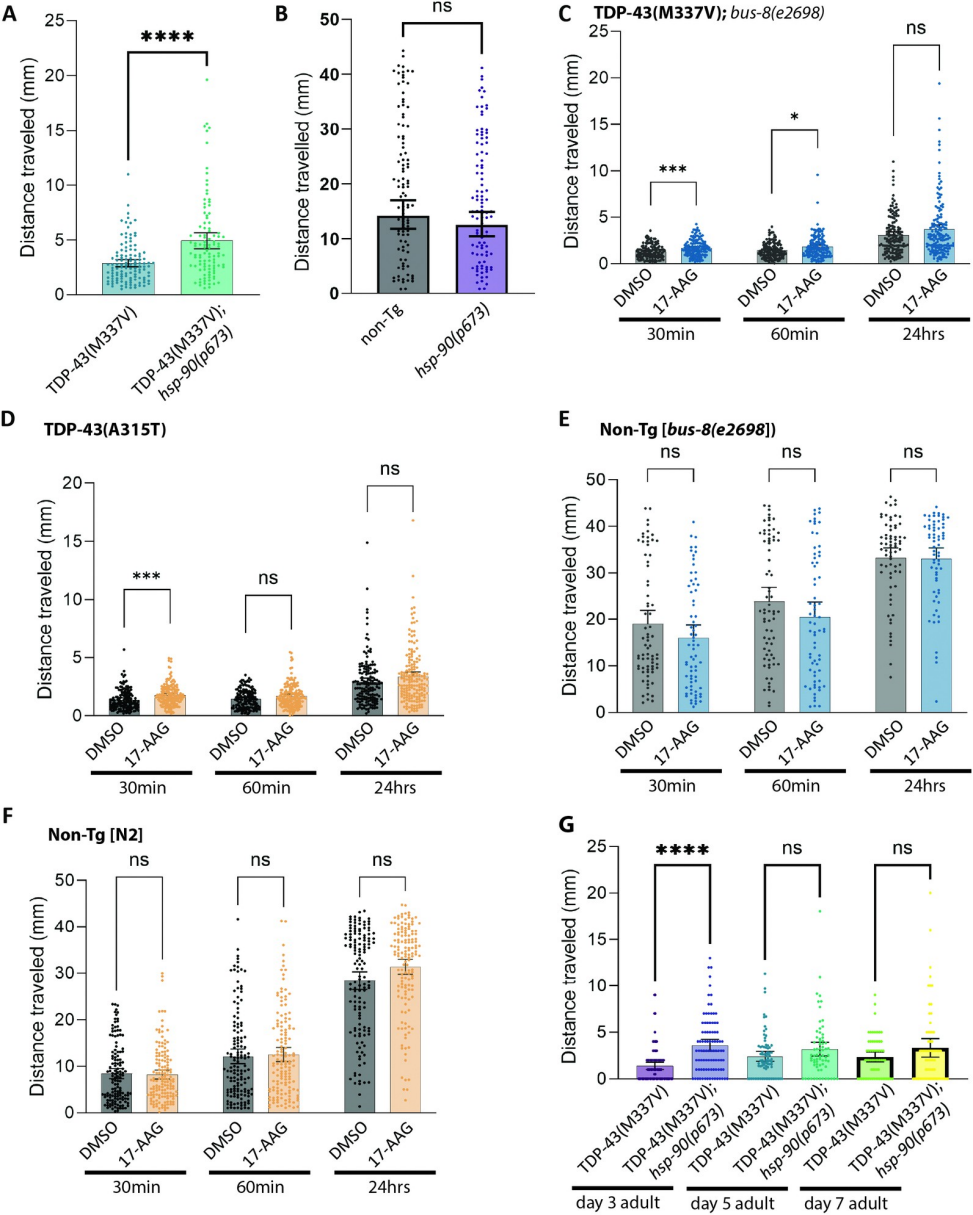
HSP-90 mutation or inhibition protects against hTDP-43 driven motor dysfunction in *C*. *elegans*. **A.** Radial locomotion assays were used to measure motor function following temperature-based inactivation of HSP-90. Animals were shifted to the restrictive temperature at L4 stage and assessed after 24 hours of HSP-90 inactivation. Animals expressing fALS TDP-43^(M337V)^ combined with the *hsp-90(p673)* mutation show an improvement in their motility. Error bars represent Mean with 95% confidence interval (CI): N = 3 independent experimental replicates; total n>100. Statistical significance as determined using Student’s t-test. (**** p<0.0001). **B.** *hsp-90(p673)* animals did not move significantly differently from wildtype (N2) animals, which were used as non-transgenic (non-Tg) controls. N = 3 independent experimental replicates; total n>100. Statistical significance as determined using Student’s t-test. (ns = not significant). **C-D.** Radial locomotion assays were used to measure motor function following pharmacological inhibition of HSP-90. Treatment with the Hsp90 inhibitor 17-AAG improves motor dysfunction caused by the expression of mutant hTDP-43 in *C*. *elegans*. Two independent transgenic strains, expressing either TDP-43^(M337V)^ **(C)** or TDP-43^(A315T)^ **(D)** exhibit improved motility after 48h of 17-AAG treatment (7.5 μM) when scored at 30 minutes, but this effect diminishes or is lost by 60 minutes or 24 hours post-removal of drug. Error bars represent Mean with 95% CI. N = 4; n>100 for the TDP-43^(M337V)^ treated worms, and n>100 when using the TDP-43^(A315T)^ strain. Statistical significance as determined using a Mixed-effects analysis (Bonferroni’s multiple comparisons test post hoc test). (*** p<0.001; *p<0.05). **E-F**. Control worms showed no difference in their motility assessment after 48h of treatment with 17-AAG (7.5 μM). Results shown combined data from multiple experiments. Error bars represent Mean with 95% CI. N = 3; n>100. Statistical significance was determined using a Mixed-effects analysis (Bonferroni’s multiple comparisons test post hoc test). **G.** Radial locomotion assays were used to measure motor function in older TDP-43^(M337V)^ at day 3, 5, or 7 of adulthood following a 24 hour temperature-based inactivation of HSP-90. TDP-43^(M337V)^;*hsp-90(p673)* animals show an improvement in their motility at day 3 of adulthood, but this is lost at days 5 and 7. Error bars represent Mean with 95% CI: N = 3 independent experimental replicates; total n>65. Statistical significance as determined using Student’s t-test. (**** p<0.0001).

To assay the post-developmental role of Hsp90 in ALS, we utilized *C*. *elegans* models which express human fALS mutant TDP-43, (TDP-43^(M337V)^, TDP-43^(A315T)^, or TDP-43^(G290A)^) pan-neuronally. These animals exhibit progressive motility defects that worsen with age, selective degeneration of motor neurons, and accumulation of phosphorylated TDP-43 [46]. We crossed *hsp-90(p673)* to TDP-43^(M337V)^, TDP-43^(A315T)^, or TDP-43^(G290A)^ transgenic strains to generate TDP-43^(M337V)^*;hsp-90(p673)*, TDP-43^(A315T)^*;hsp-90(p673)*, and TDP-43^(G290A)^*;hsp-90(p673)* strains. Multiple TDP-43 transgenic strains were used to ensure results were not due to transgene insertion site or background genotype effects in a single strain. In order to evaluate motor capacity of these animals, we shifted late larval development (L4) stage animals grown at the permissive temperature, 16°C, to the *hsp-90(p673)* restrictive temperature, 25°C, for 24 or 48 hours, and assessed their movement using a radial locomotion assay. As controls, we also tested animals expressing only *hsp-90(p673)* and the non-transgenic wildtype strain N2. We found that *hsp-90(p673)* was able to improve motility of TDP-43^(M337V)^, TDP-43^(A315T)^, and TDP-43^(G290A)^ animals (Figs 1A and S1A–S1C Fig). Both control strains, *hsp-90(p673)* and the non-transgenic strain N2, were not significantly different from one another in the radial locomotion performance (Fig 1B), indicating that *hsp-90(p673)* does not have hyperactive motility driving its apparent rescue of TDP-43 transgenic strain phenotypes. In the absence of a shift to the restrictive temperature, *hsp-90(p673)* did not suppress TDP-43 motility defects (S1D Fig).

To confirm that loss of function of HSP-90 was responsible for protection against TDP-43 driven neurotoxicity, we conducted a pharmacological assay using the Hsp90 inhibitor tanespimycin (17-allyamino-17-demethoxygeldamycin (17-AAG)), a geldamycin synthetic analog with a less toxic profile [47, 48]. To this purpose, we selected two *C*. *elegans* strains carrying either fALS mutant TDP-43^(M337V)^ or TDP-43^(A315T)^. For the TDP-43^(M337V)^ strain, in addition to the TDP-43 mutation, the animals carried a *bus-8(e2698)* mutation which disrupts the outer cuticle of *C*. *elegans*, and may increase permeability to the Hsp90 inhibitor [49]. Animals were treated with 17-AAG 7.5μM from egg throughout development. Their motility was then tested using the radial locomotion assay. Both strains showed significant improvement of the distance traveled at 30 minutes after removal from the drug (Fig 1C and 1D). This effect is lost with increased time away from the drug treatment (60 minute and 24 hour post-removal motility time points). There were relatively similar responses to 17-AAG in both Fig 1C and 1D, indicating the *bus-8(e2698)* mutation may not appreciably change uptake of 17-AAG. We did not see an effect in non-transgenic worms’ motility, either in wild-type (N2) or *bus-8(e2698)* animals, after 48h of 17-AAG treatment (Fig 1E and 1F), suggesting that baseline motility is unaffected at this dose, and that the increased movement observed in fALS model animals are not due to hyperactivity or changes in food motivation. We also tested whether treatment with 17-AAG could further improve TDP-43^(M337V)^*;hsp-90(p673)* motility. However, there was no significant difference in motility between control versus 17-AAG treated TDP-43^(M337V)^*;hsp-90(p673)* (S1E Fig), indicating that the protective phenotype of 17-AAG in TDP-43^(M337V)^ animals is likely to be through targeting HSP-90 rather than from additional off-target effects. These results are consistent with what we observed by genetic modulation of *hsp-90*, indicating loss of function of *hsp-90* protects against TDP-43 driven neurogenic motor dysfunction in adult *C*. *elegans* models of ALS.

*hsp-90* loss of function improves neuronal function in young adult animals (day 1 of adulthood, Fig 1A). However, ALS is typically a mid- to late-life disease. To test whether *hsp-90(p673)* can protect against TDP-43 in older animals, we shifted TDP-43^(M337V)^*;hsp-90(p673)* to the restrictive temperature (25°C) for 24 hours prior to testing motility at days 3, 5, or 7 of adulthood. We found *hsp-90* loss of function significantly improves TDP-43^(M337V)^ at day 3 of adulthood, but not at older ages (days 5 and 7 of adulthood) (Fig 1G), indicating there is a temporal window beyond which targeting HSP-90 is not effective.

### *hsp-90* mutation protects against neurodegeneration in a *C*. *elegans* model of amyotrophic lateral sclerosis

**Fig 2:**
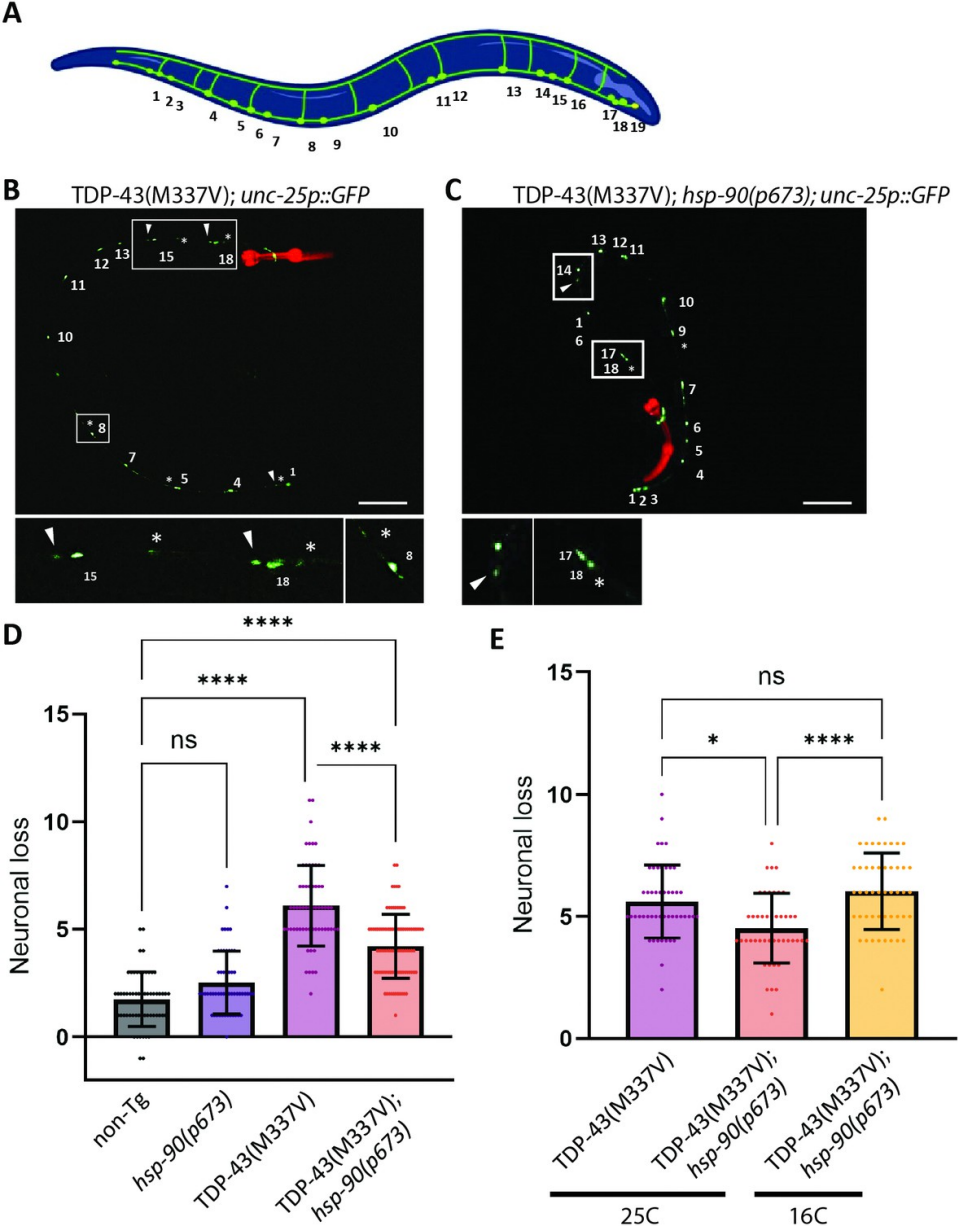
*hsp-90* mutation rescues neurodegeneration observed in TDP-43^(M337V)^ transgenic animals. GABAergic neurons marked by GFP (*unc-25p*::*GFP*) were counted in adult transgenic animals after a 24h incubation at 25°C. **A**. Cartoon model shows the positions of 19 GABAergic neurons scored using the GFP reporter. Fig 2A was created in BioRender (2024) https://BioRender.com/g76d973. **B-C.** Representative images from **B.** TDP-43^(M337V)^ and **C.** TDP-43^(M337V)^;*hsp-90(p673)* animals. White arrows indicate degenerating neurons; white stars indicate dead neurons. Scale bar = 75μm. **D**. Quantification of neuronal loss following a shift to the restrictive temperature of 25°C. Neurons were counted and the total number was subtracted from 19, the average number in a developmentally normal wild-type animal, to obtain the number of neurons lost. Some animals were scored with greater than 19 neurons, which may represent developmental variants or GFP artifacts, and resulted in a negative number of neurons lost. Error bars represent SEM: N = 3; n = 58–61. **E**. Quantification of neuronal loss without a shift to the restrictive temperature. Error bars represent SEM: N = 2; n = 45–49. Statistical significance was determined using a non-parametric Kruskal-Wallis test (Dunn’s multiple comparisons post hoc test). (**** p<0.0001; **p<0.01).

ALS model *C*. *elegans* develop a normal complement of motor neurons and are similar to wild-type animals at L4 stage, but exhibit degeneration of motor neurons beginning at day 1 of adulthood [46,50]. To test whether loss of HSP-90 could prevent this neurodegeneration, we utilized a reporter strain expressing GFP in GABAergic motor neurons. We shifted late larval development (L4) stage TDP-43^(M337V)^*;hsp-90(p673)* grown at 16°C to the restrictive temperature 25°C for 24 hours, and assessed their number of neurons as day 1 adult stage-matched animals. We found an increase in GABAergic neuronal survival after inactivation of HSP-90 (Fig 2A–2D), indicating an amelioration of the neurodegenerative process that occurs in our model of TDP-43 proteinopathy with age. Without the temperature shift, there was not observable protection against neuron loss in TDP-43^(M337V)^*;hsp-90(p673)* (Fig 2E).

### Loss or inhibition of HSP-90/ Hsp90 decreases total and phosphorylated TDP-43

**Fig 3:**
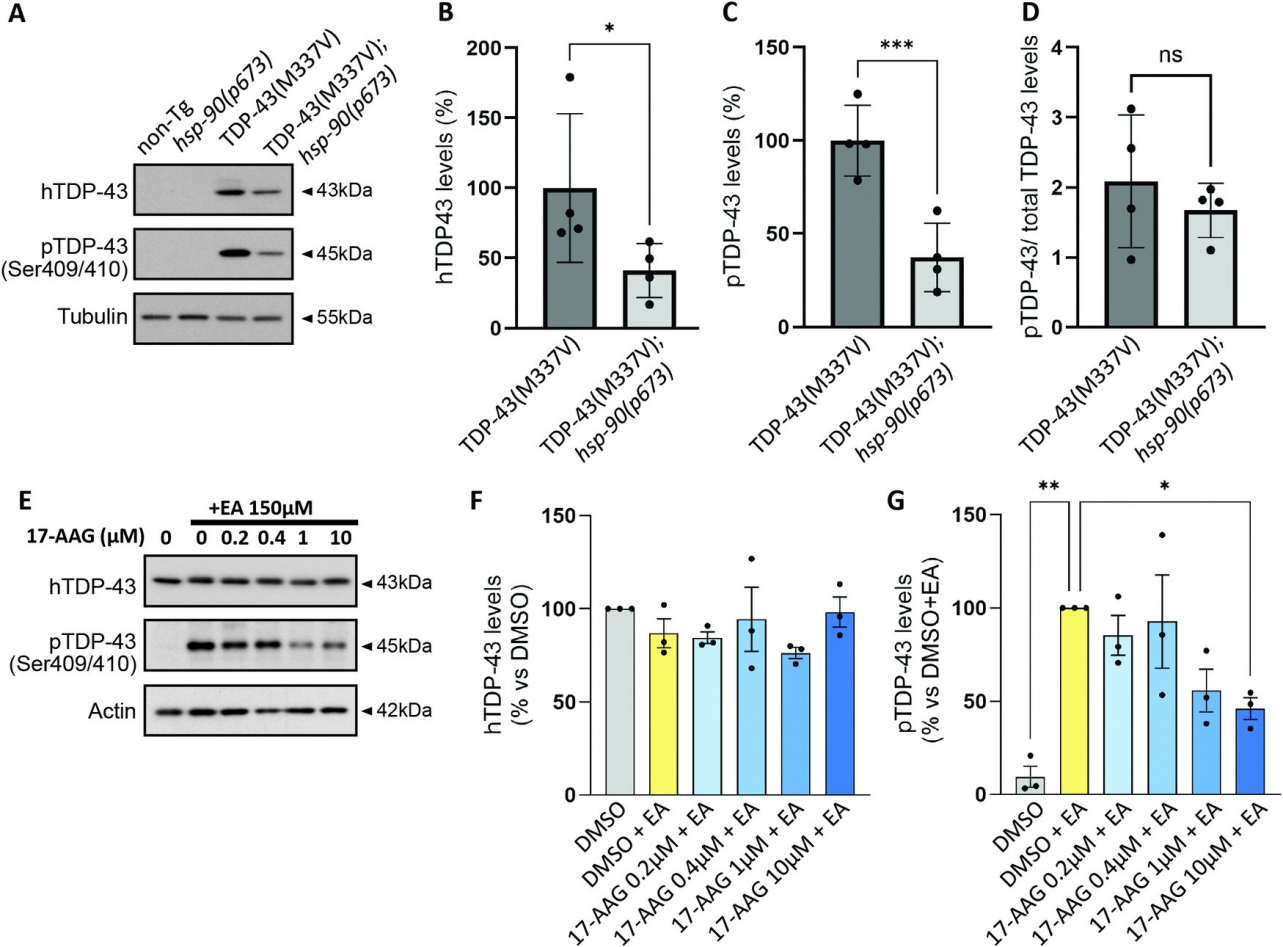
Hsp90 mutation or inhibition decreases accumulation of phosphorylated TDP-43 **A.** Representative immunoblots for total and phosphorylated TDP-43 in *C*. *elegans* samples**. B-C.** Mutant *hsp-90* significantly decreases the levels of total and phosphorylated species of TDP-43 (pTDP-43). Error bars represent SEM: N = 4. Statistical significance as determined using unpaired t-test. (*p<0.05, **p< 0.01) **D.** The ratio of phosphorylated TDP-43 to total TDP-43 (pTDP-43/ total TDP-43) is unchanged in TDP43^(M337V)^ versus TDP43^(M337V)^;*hsp-90(p673)*. **E.** Representative immunoblots for endogenous total and phosphorylated TDP-43 in HEK293T cells pre-treated with increasing amounts of the Hsp90 inhibitor 17-AAG and exposed to Ethacrynic acid 150μM. **F-G.** Quantification of replicate immunoblots shows no significant differences in total TDP-43 protein levels but a trend towards a reduction in the levels of pTDP-43 in a dose-dependent manner, reaching statistical significance at the highest dose tested, 10μM. Results combined data from multiple experiments. Error bars represent SEM. N = 3. Statistical significance was determined using the One-way ANOVA test (Dunnett’s multiple comparisons post hoc test) (* p<0.05).

Because Hsp90 has a direct role in modulating TDP-43 misfolding and toxicity [27, 31, 42] we asked whether *hsp-90* loss of function affected accumulation of total or disease-associated phosphorylated TDP-43 (pTDP-43) proteins in *C*. *elegans*. Consistent with our behavioral findings, we observed a dramatic decrease in both total and pTDP-43 protein levels following a 24 hour shift to the restrictive temperature (Fig 3A–3C). However the ratio of pTDP-43 to total TDP-43 remains unchanged suggesting a general lowering of TDP-43 rather than selective targeting of one protein species (Fig 3D). We do not see differences in total or pTDP-43 maintained at the permissive temperature in the absence of a shift to the restrictive temperature (S2A–S2D Fig). We also tested mRNA expression levels of the transgene at both the permissive and restrictive temperatures. However, we found no significant differences in mRNA levels, suggesting decreased TDP-43 protein at the restrictive temperature is downstream of transcription (S2E and S2F Fig). These data may indicate Hsp-90 protein normally interacts with and stabilizes TDP-43 in our transgenic model of TDP-43 proteinopathy, thus participating in protein accumulation and hyperphosphorylation.

To further evaluate whether Hsp90 inhibition has conserved effects against accumulation of phosphorylated TDP-43, we employed a cell culture model of TDP-43 proteinopathy. This model utilizes acute exposure to Ethacrynic acid (EA), a cytotoxic agent, to stimulate TDP-43 phosphorylation, likely through a glutathione depletion-mediated increase in cellular oxidative stress [51,52]. HEK293T cells were pre-treated with the Hsp90 inhibitor tanespimycin (17-allyamino-17-demethoxygeldamycin (17-AAG) 24h prior to stimulation with EA for 3 hours. We found exposure to EA induced an increase in phosphorylated TDP-43 levels in HEK293T cells, and the treatment with increasing doses of 17-AAG exerted a protective role, gradually reducing phosphorylated TDP-43 levels in a dose-dependent manner without affecting total TDP-43 (Fig 3E–3G).

### *hsp-90* mutation does not increase proteasome activation

**Fig 4:**
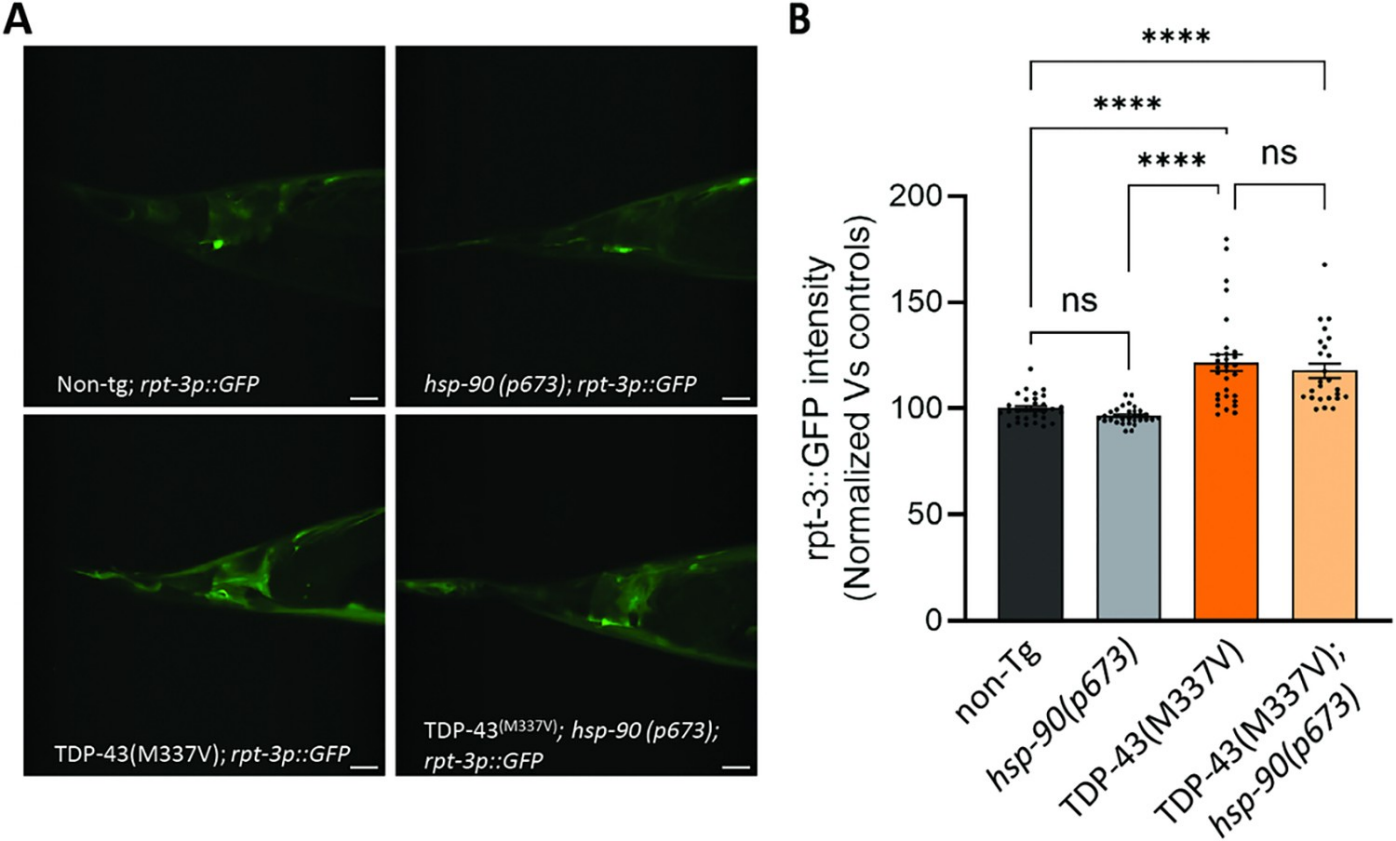
*hsp-90* mutation does not increase proteasome activation. **A.** Representative photomicrographs of proteasome reporter *rpt-3p*::*GFP* near the tail of non-Tg, *hsp-90(p673)*, TDP43^(M337V)^, and TDP43^(M337V)^;*hsp-90(p673)* animals. Scale bars = 10μm. **B.** TDP-43^(M337V)^ expressing *C*. *elegans* increase *rpt-3p*::*GFP* reporter expression, but *hsp-90* mutation does not affect reporter expression either independently or in combination with TDP-43^(M337V)^. Error bars represent SEM: N = 3, n = 25–31. Statistical significance as determined using the non-parametric Kruskal-Wallis test (Dunn’s multiple comparisons post hoc test).

Inhibiting Hsp90 binding destabilizes its client proteins, leading to their degradation by the proteasome [53]. Thus, it is possible the reduced TDP-43 protein observed in TDP-43^(M337V)^;*hsp-90(p673)* results from an increase in proteasome-mediated degradation. To explore whether Hsp90 inactivation increases proteasome activity in *C*. *elegans*, we generated new transgenic strains by crossing our TDP-43^(M337V)^, *hsp-90(p673)*, and TDP-43^(M337V)^;*hsp-90(p673)* strains with a proteasome reporter strain carrying an *rpt-3* promoter-driven GFP [54]. The *rpt-3p*::*GFP* transcriptional reporter exhibits increased fluorescence under conditions that cause increased proteasome biogenesis, including accumulation of misfolded or aggregation-prone proteins [54, 55]. We observed an increase in *rpt-3p*::*GFP* fluorescence intensity in TDP-43^(M337V)^-expressing animals comparable to previously published *rpt-3p*::*GFP* activators [54, 55], indicating fALS associated TDP-43 triggers increased proteasome biogenesis (Fig 4A and 4B). In contrast, the *hsp-90(p673)* mutation has no effect on rpt-3p::GFP fluorescence on its own (Fig 4A and 4B). Moreover, the *hsp-90* mutation did not modulate the proteasome activation caused by TDP-43^(M337V)^. Altogether, these data suggest that TDP-43^(M337V)^ expression leads to an increase in proteasome gene expression, but this phenomenon is not regulated by *hsp-90*. Thus, the reduction in total and phosphorylated TDP-43, improvement in motility, and reduced neurodegeneration that we observe in in TDP-43^(M337V)^;*hsp-90(p673)* animals is likely not driven by a change in proteasome levels.

### The small heat shock protein HSP-16.1 is required for HSP-90 loss of function mediated TDP-43 suppression

**Fig 5:**
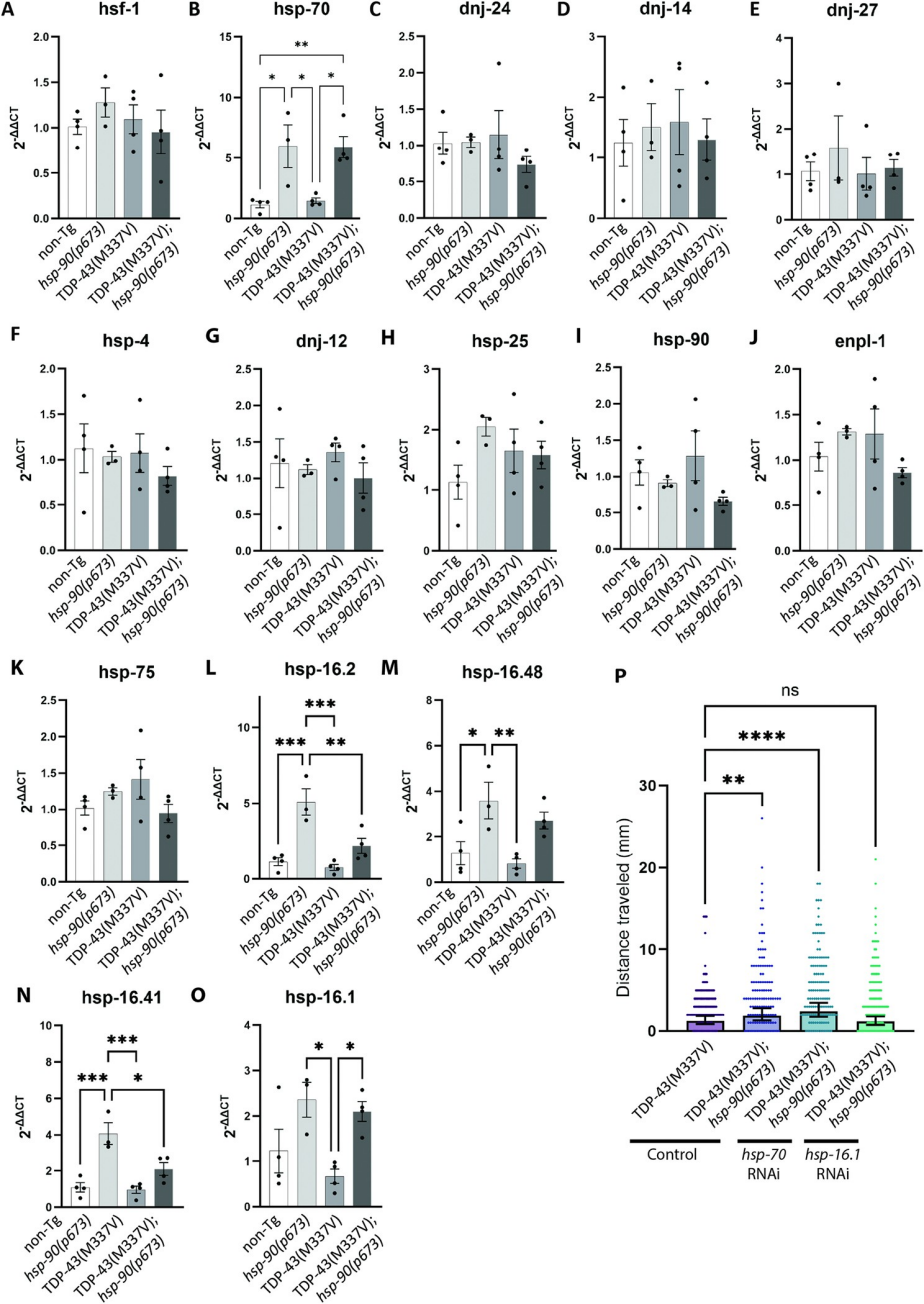
*hsp-90* mutation selectively increases heat shock protein expression **A-O.** qRT-PCR was used to detect mRNA expression changes in heat shock proteins of interest. No significant expression differences were observed for *hsf-1* **(A)**, *dnj-24* **(C)**, *dnj-14* **(D)**, *dnj-27* **(E)**, *hsp-4* **(F)**, *dnj-12* **(G)**, *hsp-25* **(H)**, *hsp-90* **(I)**, *enpl-1* **(J)**, *hsp-75* **(K)**. Both *hsp-70* **(B)** and *hsp-16*.*1* **(O)** have increased expression in *hsp-90(p673)* and TDP43^(M337V)^; *daf-21(p673)* animals. *hsp-16*.*2* **(L)**, *hsp-16*.*48* **(M)**, and *hsp-16*.*41* **(N)** were increased in *hsp-90(673)* animals, but this expression increase was attenuated in TDP43^(M337V)^; *daf-21(p673)*. Error bars represent SEM. N = 3–4; Statistical significance as determined using the One-way ANOVA test (Tukey’s multiple comparisons post-hoc test) or a non-parametric Kruskal-Wallis test (Dunn’s multiple comparisons post hoc test) when required. (*p<0.05; **p<0.01; *** p<0.001). **P.** TDP43^(M337V)^ and TDP43^(M337V)^;*hsp-90(p673)* were treated with control (empty vector), *hsp-70*, or *hsp-16*.*1* targeting RNAi via feeding exposure starting at hatching (L1 stage). At L4 stage, animals were shifted to the restrictive temperature (25°C) for 24 hours. Radial locomotion assays were used to measure motor function. Error bars represent Mean with 95% CI: N = 3 independent experimental replicates; total n>65. Statistical significance as determined using One-way ANOVA test (Tukey’s multiple comparisons post-hoc test). (** p<-.-1, **** p<0.0001, ns = not significant).

Hsp90 has a wide variety of clients, including other heat shock proteins and key players regulating the heat shock response. In order to evaluate how this protein homeostasis network may respond to altered HSP-90 function, we determined the mRNA levels of several elements of the heat shock response in the TDP-43^(M337V)^*;hsp-90(p673)* strain after a 24 hour shift to the restrictive temperature (25°C), including the Hsp90 clients *hsf-1*, the major transcription factor for heat shock proteins [56,57], *hsp-70*, an Hsp90 co-chaperone, the Hsp70 family member *hsp-4/* HSPA5, and the heat shock protein *hsp-*25/ HSPB8. We also tested Hsp40 family members *dnj-24*/ DNAJB8, and *dnj-14*/ DNAJC5, which are chaperones involved in muscle maintenance and proteostasis [37,58,59], and *dnj-27*/ DNAJC10, *dnj-12/* DNAJA1, which are involved in the protection against proteotoxicity in the protein unfolded response [60–62]. In addition we checked the levels of *hsp-90*, *hsp-75*, and *enpl-1*. Interestingly, of the 15 heat shock protein-related elements analyzed, only *hsp-70* mRNA levels were affected by the *hsp-90* mutation (Fig 5). *hsp-70* mRNA levels are increased in *hsp-90(p673)* mutants (Fig 5A–5K). This chaperone has been described as working in concert with Hsp90 in the regulation of protein toxicity in numerous neurodegenerative diseases [16,63–65]. Additionally, we also tested mRNA levels of four small heat shock proteins from the Hsp16 chaperone family, homologs of the human α/β crystallin proteins. These small heat shock proteins are inducible under cellular stress conditions, including heat shock, oxidative stress, and hypoxia [66–69]. We observed an increase in the mRNA levels of *hsp-16*.*2*, *hsp-16*.*48*, *hsp-16*.*41*, and *hsp-16*.*1* in the *hsp-90(p673)* mutant animals versus wild-type (non-transgenic) animals, and there was a trend towards upregulation of *hsp-16*.*2*, *hsp-16*.*48*, and *hsp-16*.*41* in the TDP-43^(M337V)^*;hsp-90(p673)* double mutants compared to TDP-43^(M337V)^ alone. This upregulation reached statistical significance in the case of chaperone *hsp-16*.*1* (Fig 5O).

Both *hsp-70* and *hsp-16*.*1* are inducible by heat stress, and it is possible that heat shock responses are contributing to protection against TDP-43 neurotoxicity. To test this, we exposed TDP-43^(M337V)^ to a short heat stress (2 hours at 34°C), a dose that has previously been shown to promote lifespan extension in *C*. *elegans* [70, 71]. We then measured motility after a 24 hour recovery period. However, heat stressed TDP-43^(M337V)^ animals moved worse than animals without heat stress (S3 Fig), suggesting *hsp-90(p673)* protection may be mediated by a non-heat shock inducible pathway. To directly examine whether HSP-70 or HSP-16.1 are required for *hsp-90* loss of function mediated improvements in TDP-43 phenotypes, we treated TDP-43^(M337V)^*;hsp-90(p673)* strain with RNAi targeting either *hsp-70* or *hsp-16*.*1*. We did not observe any change in *hsp-90(p673)* protection in animals treated with *hsp-70*, indicating that HSP-70 may not be required for this effect (Fig 5P). However, we found that RNAi targeting *hsp-16*.*1* was sufficient to prevent *hsp-90(p673)* mediated protection against TDP-43 (Fig 5P). Taken together, these data suggest that HSP-16.1 functions downstream of HSP-90 loss of function to enable protection against fALS TDP-43.

## Discussion

Protein level regulation during cellular stress conditions is essential to maintain homeostasis and reduce cellular toxicity. In neurodegenerative proteinopathies, these regulatory mechanisms can be impaired, resulting in the accumulation of neurotoxic misfolded proteins in disease-affected regions of the central nervous system [72]. The chaperone Hsp90 plays a crucial role in the quality control of proteins, including preventing protein misfolding and assisting in protein degradation, which counteracts protein aggregation [27,28]. However, its contribution to disease is not fully understood since both protective and deleterious actions have been described in different experimental models of neurodegeneration [12,42].

In the present study, we report that HSP-90 promotes disease in a transgenic *C*. *elegans* fALS model expressing human mutant TDP-43. Either by genetic intervention or pharmacological inhibition, using Tanespimycin (17-AAG), we partially rescue neuronal function in our models of TDP-43 proteinopathy (Figs 1A, 1C and 1D and S1A–S1C Fig). Both 17-AAG and genetic inactivation of HSP-90 have a similar mechanism of action: the *hsp-90* mutant has a point mutation at the C-terminus that induces a drastic charge change likely affecting protein function [39] and reduced ATP turnover ([73], and 17-AAG inhibitor binds Hsp90 N-terminally, acting as a competitive inhibitor for the ATP binding site ([74]. We also show that genetic inactivation in *C*. *elegans* or pharmacological inhibition of Hsp90 in HEK293 cells partially reduces phosphorylated TDP-43 levels, indicating a conserved role in mediating TDP-43 protein dynamics (Fig 3C and 3F [51, 75]). Our results have been confirmed by three different experimental approaches; whether we tested Hsp90 chaperone dysfunction or inactivation *in vivo* or *in vitro*, using different experimental models, we show a protective effect against TDP-43 related pathology by reducing Hsp90 activity. Interestingly, we find that Hsp90 inactivation only protects against TDP-43 in younger adult *C*. *elegans* (days 1 or 3 of adulthood) (Fig 1G). These data suggest that Hsp90 loss of function may protect best early in disease, potentially linked to the increasing loss of neurons observed over time in this model [46]. Efforts to translate Hsp90 inhibition to mammalian models as a therapeutic strategy will need to consider potential temporal windows to target Hsp90 during disease progression or aging.

Consistent with the improvement in animal motility, our results show that dysfunction of the HSP-90 chaperone in TDP-43^M337V^;*hsp-90(p675)* animals produces a neuroprotective effect on GABAergic motor neurons (Fig 2A and 2B), an effect that we did not observe when animals were maintained at their growth temperature (16°C) without inactivation of HSP-90 (Fig 2C). GABAergic motor neurons are especially vulnerable in this experimental model [46], indicating that blocking HSP-90 activity results in a strong neuroprotective effect.

Hsp90 is a potential target to prevent protein accumulation in multiple neurodegenerative proteinopathies, including tauopathies, amyloid beta [76,77], poly-glutamine repeat expansion disorders, and Parkinson’s disease [78–80]. Increased levels of Hsp90 have been described in the course of Parkinson’s disease, forming protein complexes with alpha-synuclein, which ultimately leads to the formation of protein aggregates in the mitochondria. When this chaperone is ubiquitylated for degradation through the UPS, there is also an increase in the clearance of alpha-synuclein [81]. In the context of Alzheimer’s disease, Hsp90 can also stabilize and maintain tau [82]. When phosphorylated, tau binds with great affinity to Hsp90/Hsp70/co-chaperone complexes, triggering the stabilization of pathological tau species. Therefore, Hsp90 inhibition strategies may dismantle this complex and reduce phosphorylated tau burden in cells [83]. Similarly, TDP-43 can form TDP-43/Hsp90/co-chaperone complexes, and dismantling this association drives TDP-43 to degradation pathways [42]. Moreover, Hsp90 inhibition can be neuroprotective in ALS, facilitating the clearance of TDP-43 by releasing the transcription factor HSF-1, which activates the heat shock response. [84].

Hsp90 inhibition has previously been shown to promote proteasome-mediated degradation of other known clients [26,33]. In order to test whether proteasomal changes underlie Hsp90-mediated TDP-43 protein accumulation or clearance, we generated a worm strain including a reporter for proteasome biogenesis in *C*. *elegans* [54]. Interestingly, we found that animals with diminished HSP-90 chaperone activity, *hsp-90(9673)*, do not exhibit altered proteasome biogenesis. However, both strains carrying the TDP-43^M337V^ transgene showed an increase in proteasome biogenesis, regardless of HSP-90 chaperone activation status (Fig 4). This indicates that while fALS associated TDP-43 triggers proteasomal changes, these changes are unlikely to be the main contributor or primary factor in the decreased protein accumulation observed in TDP-43^(M337V)^;*hsp-90(p673)*. This is the first report in *C*. *elegans* models showing that TDP-43^(M337V)^ expression leads to likely proteasome activation, but other publications have shown similar results for animals expressing amyloid beta, another neurodegenerative disease associated aggregating protein [54]. Importantly, these data do not exclude the possibility that other components of the ubiquitin proteasome system, such as ubiquitin ligases or ubiquitin-binding proteins, contribute to the protective effect of Hsp90 inhibition through enhanced TDP-43 clearance [85,86]. In addition to participating in the ubiquitin proteasome degradation pathway, Hsp90 also interfaces with autophagy mediated protein degradation [87]. In fact, depending on which co-chaperone is interacting with Hsp90, there is a preference for the degradation pathway that clients go through [33,42]. Moreover, the clients of Hsp90 include proteins involved in autophagy processes, such as AGT proteins, ULK1, or cargo recognition proteins in lysosomes, such as LAMP-2α [14]. Therefore, based on our results, autophagy rather than the proteasome may control clearance of phosphorylated TDP-43 when HSP-90 activity is abrogated, although future experiments will need to confirm this hypothesis.

Chaperone-mediated autophagy (CMA) is an essential protein quality control system, and alterations in CMA may contribute to neurodegenerative diseases of aging [88,89]. Several studies have shown neuroprotection from enhancing CMA, and the use of activators of this degradation pathway has been proposed for clinical trials [90–94]. Our data suggest this may be a viable approach to reduce phosphorylated TDP-43 in ALS and FTLD-TDP.

In the present work, we have observed how genetic disruption of *hsp-90* leads to an increase in *hsp-70* mRNA levels (Fig 5B), which could contribute to the neuroprotective effect in our model of fALS, although RNAi targeting *hsp-70* did not modify *hsp-90* loss of function impacts on TDP-43 phenotypes (Fig 5P). Hsp90 inhibition releases the transcription factor Hsf1, which may drive neuroprotection through the induction of molecular chaperones, especially Hsp70 [95,96]. Moreover, Hsp90 inhibition allows longer transcriptional activation times of Hsp70 [56]. In agreement with our results, other studies have found beneficial effects from the reduction of Hsp90 together with a specific increase of the chaperone Hsp70 [96,97]. In *C*. *elegans*, HSP-90 modulates proteostasis by sequestering HSF-1, preventing it from raising the turnover of proteins, and protecting new and mature proteins from being degraded under physiological conditions [98]; however, when cells are under stress, HSP-90 is dissociated from HSF-1 in order to activate the heat shock response, which includes the upregulation of *hsp-70*. Hsp70 induces protein degradation by activating both autophagy and the ubiquitin proteasome system pathways [99]. In fact, RNA-binding proteins including TDP-43 and Fus may be cleared by chaperone-mediated autophagy [100]. However, future studies are needed for in depth investigation of this pathway in ALS.

In addition to Hsp70, Hsp90 inhibition induces the overexpression of another small heat shock protein, alpha β crystallin [101]. Under physiological conditions, this protein forms multimeric complexes that sequesters them with minimal chaperone activity. However, during stress stimulus and disease, these protein complexes break down, increasing the accessibility of crystallins to interact with aggregation-prone proteins such as SOD1 or tau, and prevent the formation of pathological aggregates [102,103]. Here, we have investigated whether HSP-90 inactivation affects the HSP-16 protein family, the main *C*. *elegans* homologs of the human α/β crystallin proteins. The HSP-16 family has been widely studied in *C*. *elegans* in association with several neurodegenerative diseases, including Alzheimer’s disease, Parkinson’s disease, and retinopathies [104–107]. These small heat shock proteins are inducible under cellular stress conditions, including heat shock, oxidative stress, and hypoxia [66–69]. We found genetic modulation of *hsp-90* increased *hsp-16* family mRNA levels (Fig 5L–5O). Increased levels of HSP-16 may contribute to the neuroprotection seen in our *C*. *elegans* fALS model. In fact, our data show that RNAi mediated reduction in *hsp-16*.*1* prevents *hsp-90* loss of function mediated protection against TDP-43 (Fig 5P). Similar to our results, other studies have shown how, in response to an upregulation of heat shock proteins from the *hsp-16* family, there is a suppression of toxicity in other neurodegenerative diseases with a protein accumulation component, such as Alzheimer’s disease and Parkinson’s disease [104–106]. Focusing on *hsp-16*.*1*, our results suggest a new mechanism by which genetic manipulation of the Hsp90 chaperone may be exerting a protective effect. HSP-16.1 is selectively localized in the Golgi apparatus and endosomes. The Golgi apparatus plays a particularly important role as a reservoir of intracellular Ca^2+^, being responsible for the control of calcium homeostasis and thus regulating cell necroptosis. Both HSP-16.1 and the major heat shock transcription factor HSF-1 participate in the regulation of proteins that control calcium homeostasis in the Golgi under conditions of cellular stress. Specifically, HSP-16.1 appears to stabilize the calcium pump PMP-1, which reduces Ca^2+^ levels in the cytoplasm, exerting a protective effect against different stimuli, including protein aggregation of α-synuclein [108].

Summarizing, our results indicate that Hsp90 participates in the pathogenesis of TDP-43 proteinopathy, potentially by stabilizing TDP-43, which contributes to its accumulation and formation of toxic aggregates. Hence, biological inactivation of Hsp90 results in a reduction in the burden of toxic species of TDP-43, which induces a neuroprotective effect by activation of the heat shock response.

## Materials and Methods

### Strains

Temperature sensitive strains and relevant controls were maintained at 16°C; other experimental strains were maintained at 20°C on nematode growth media (NGM) agar plates with OP50 *E*. *coli* as a food source. N2 (Bristol) was used as wild-type *C*. *elegans*. Transgenic strains used were CK423 *bkIs423[Psnb-1*::*hTDP-43(M337V)+Pmyo-2*::*dsRED]*, CK426 *bkIs426[Psnb-1*::*hTDP-43(A315T)+Pmyo-2*:: *dsRED]*, CK422 *bkIs422[Psnb-1*::*hTDP-43(G290A)+ Pmyo-2*::*dsRED]* [46], CK443 *bkls443*[*bus-8(e2698); Psnb-1*::*TDP-43 (M337V)+Pmyo-2*::*dsRED*] [50]. CB6055 *bus-8(e2698)*, and JT6130 *hsp-90(p673)* strains were obtained from the *C*. *elegans* Genetics Center (CGC). GR2183 *mgIs72[rpt-3p*::*gfp]* was a gift from Dr. Nicolas Lehrbach [54].

### Construction of double and triple mutant strains

To generate NLS56 *bkIs423[Psnb-1*::*hTDP-43(M337V)+Pmyo-2*::*dsRED];hsp-90(p673)*, referred to in the paper as TDP-43^(M337V)^*;hsp-90(p673)*, NLS95 *bkIs426[Psnb-1*::*hTDP-43(A315T)+Pmyo-2*:: *dsRED]*; *hsp-90(p673)* referred to in the paper as TDP-43^A315T^;*hsp-90(p673);*, and NLS94 *bkIs422[Psnb-1*::*hTDP-43(G290A)+ Pmyo-2*::*dsRED];hsp-90(p673)*, referred to in the paper as TDP-43^(G290A)^;*hsp-90(p673)*, CK423, CK426 and CK422 heterozygous males were mated to JT6130 hermaphrodites, and F1 progeny were picked to individual plates. F2 animals were singled and their progeny were examined to identify populations homozygous for both *hTDP-43* transgenes (as detected by 100% population expression of the fluorescent reporter transgene *Pmyo-2*::*dsRED*) and displaying the JT6130 phenotype (100% Dauer arrest at 25°C). For the neurodegeneration assays, NLS56 and JT6130 were crossed to the GABAergic neuron reporter strain CZ1200 *juIs76[unc-25p*::*GFP* + *lin-15(+)*; *lin-15B&lin-15A(n765)]* to generate strains NLS73 and NLS74. For the proteasome reporter strains, NLS56, CK423 and JT6130 strains were mated with GR2183 males.

### Behavioral analysis

Radial locomotion assays were performed as described in [109] with modifications. L4 stage-matched worms were shifted from the permissive *hsp-90(p673)* growth temperature 16°C to the restrictive temperature 25°C for 24h prior to the test. Worms were then picked onto 100 mm nematode growth medium (NGM) agar plates covered with a uniform bacterial lawn and allowed to move freely. Worm location was annotated after 30 minutes or 24 hours, and dispersal from the starting position was measured. Figures show results from at least three independent replicates, and statistical analyses were performed using GraphPad Prism software.

### Live-mount fluorescence microscopy for the proteasome reporter imaging

Proteasome subunit upregulation was visualized by the expression of GFP under the *rpt-3* proteasome subunit gene promoter in GR2183, NLS56, JT6130 and CK423 strains. Live worms were mounted as described in [110] with modifications. Worms were mounted on 4% agarose and immobilized with 500nM sodium azide. L4 stage-matched worms were shifted to the restrictive temperature for *hsp-90(p673)* for 24h at 25°C prior to live mounting. Images were acquired using a Nikon A1R (Nikon USA, Melville, NY) confocal microscope using 100x oil immersion objective. Figures show results from at least three independent replicates, and statistical analyses were performed using GraphPad Prism Software.

### GABAergic neuron loss assay

GABAergic inhibitory motor neurons were visualized by the expression of GFP under the *unc-25* promoter (*unc-25p*::*GFP*) in CZ1200, CK473, NLS73 and NLS74 transgenic animals. Live worms were mounted as described above. L4 stage-matched worms were shifted to the restrictive temperature for *hsp-90(p673)* for 24h at 25°C prior to live mounting. To assay GABAergic neuron loss in Day 1 adult worms, a subset of VD and DD GABAergic neurons were scored under fluorescent microscopy using an Olympus 40× dry objective and a 60× oil objective on the DeltaVision Elite (Applied Precision) system. The number of live neurons was counted blinded to genotype. Figures show results from at least three independent replicates. Statistical analyses were performed using GraphPad Prism software. Photomicrographs of live worms were obtained using a 20x objective in a Leica DM6 microscope with a DFC 7000 digital camera (Leica Microsystems).

### Real time qRT-PCR analysis

Approximately 10,000 stage-matched *C*. *elegans* were grown at 16°C to L4, and then shifted to 25°C for 24 hours until day 1 adult. They were then washed off plates with M9 buffer, collected by centrifugation, and snap-frozen in liquid nitrogen, and stored at −70°C. Total RNA was extracted from *C*. *elegans* samples using TRI-Reagent (Thermo Fisher Scientific, Inc.) by manufacturer’s recommendations. The total amount of RNA extracted was quantified by spectrometry at 260 nm, its purity was evaluated by the ratio between the absorbance values at 260 and 280 nm, and its integrity was confirmed by agarose gel electrophoresis to detect ribosomal RNA (rRNA) bands and lack of smearing. Single-stranded complementary DNA (cDNA) was synthesized from 1 μg of RNA using the commercial kit iScript gDNA Clear cDNA Synthesis Kit (Bio-Rad, CA, USA). Quantitative real-time PCR assays were performed using the iTaq Universal SYBR Green Supermix kit (Bio-Rad, CA, USA) on a CFX Connect Real-Time PCR Detection system (BioRad, CA, USA) and primers in S1 Table. Data were normalized within samples using an internal reference control gene (*iscu-1*). The threshold cycle (CT) was calculated by the instrument’s software (CFX Maestro Software for CFX Real-Time PCR instruments. BioRad, CA, USA). Expression levels were calculated using the 2-ΔΔCt method [111], but, for presentation, data were transformed to the part per unit over the mean obtained in the wild-type group for each parameter. Each experimental group was tested in three to five biological replicates and two technical replicates.

### Immunoblotting worm sample preparation

Approximately 10,000 stage-matched day 1 adult *C*. *elegans* were washed off plates with M9 buffer, collected by centrifugation, and snap-frozen in liquid nitrogen, and stored at −70°C. Then, protein was extracted by resuspending pellets in 1× SDS protein sample buffer (0.046 M Tris, 0.005 M EDTA, 0.2 M dithiothreitol, 50% sucrose, 5% sodium dodecyl sulfate, 0.05% bromophenol blue), sonicated, boiled for 10 min at constant temperature (96° C), and stored at -20°C.

### Immunoblotting and quantitation

Samples were loaded and resolved on 4–15% precast criterion sodium dodecyl sulfate (SDS) polyacrylamide gel electrophoresis gradient gels and transferred to PVDF membrane as recommended by the manufacturer (Bio-Rad). On immunoblots, human TDP-43 was detected by a monoclonal antibody, anti-TDP-43 (ab57105[2E2-D3], Abcam, 1:10,000). TDP-43 phosphorylated at pS409/S410 was detected by a monoclonal antibody, anti-phospho TDP-43 (pS409/410, TIP-PTD-M01, Cosmobio, 1:1,000)., and Hsp70 was detected by a polyclonal antibody, HSPA1A (GTX111088, GeneTex, 1:15,000). *C*. *elegans* β-tubulin levels were measured using monoclonal antibody E7 (Developmental Studies Hybridoma Bank, 1:5000) as a loading control. HRP-labeled goat anti-mouse IgG secondary antibody (Jackson ImmunoResearch, 115-005-003) was used at a dilution of 1:5000. HRP-labeled mouse anti-rabbit IgG secondary antibody (Jackson ImmunoResearch, 211-005-109) was used at a dilution of 1:2,500. Quantitation was completed by ImageJ software densitometry analysis of scanned film images.

### Small molecule inhibitor assay

To determine dosing for Tanespimycin (17-N-allylamino-17-demethoxygeldanamycin, 17-AAG) assays, we performed a dose response curve in both N2 and *bus-8(e2698)* testing 0.1, 1, 2, 5, 7.5, 10, 20, 50, 100, and 150μM 17-AAG. Treated animals were monitored for developmental delays, developmental arrest, and normal movement. We found that 7.5μM was the highest dose that avoided obvious negative consequences for treated animals, and therefore this dose was chosen for additional experiments. 2 mL of nematode growth medium (NGM) were added into 35 X 10mm Petri dishes (Corning, NY, USA). Each plate was then seeded with 200 μL of 10x concentrated OP-50 *E*. *coli*. After the bacterial lawn dried, bacteria were killed by UV irradiation to minimize metabolism of test compounds. 40 μL of Tanespimycin (17-N-allylamino-17-demethoxygeldanamycin, 17-AAG) (Cayman Chemical, USA) 375 μM dissolved in 0.2% DMSO (Sigma-Aldrich, MO, USA) were added to each plate, to reach a final concentration of 7.5μM. Approximately 100–150 eggs were seeded and grown for 3 days at 20°C, when L4- 1 day adult worms were assessed for locomotory defects as described above. C. *elegans* strains N2, CK426, CB6055, and CK443 were used for this screening. The cuticle-defective *bus-8(e2692)* mutation is included to enhance small molecule entry into the animals [49). Four independent replicates were tested with two technical replicates each time.

### Cell culture and treatment

Cultured HEK293T cells were maintained in Dubecco’s Modified Eagle’s Medium (DMEM, Corning, NY, USA) supplemented with 10% fetal bovine serum (FBS, Sigma-Aldrich, MO, USA) and antibiotics (Thermo Fisher Scientific, Inc.) in 5% CO_2_ and 37°C. For experiments, cells were plated on 6 well dishes and incubated until reached 80% confluence. Then, cells were treated with 0.2, 0.4, 1, and 10μM of Tanespimycin (17-N-allylamino-17-demethoxygeldanamycin, 17-AAG) diluted on DMSO (Cayman Chemical, USA). 24h later, cells were incubated with Ethacrynic acid at 150μM for 3h. Then, cells were washed with cold-PBS and harvested for Immunoblotting.

### Heat shock assay

L4 stage-matched worms were shifted from 20°C to 35°C for 2 hours. They were then returned to 20°C for 24 hours, before performing radial locomotion assays as described in the Methods (behavioral analysis).

### RNA interference (RNAi)

Bacteria expressing RNAi constructs were purchased (Horizon Discovery, USA) and gene targeting insert validated by sequencing. RNAi bacteria were seeded onto NGM plates supplemented with 2mM isopropyl-b-D-thiolgalactophyranoside (IPTG). The following day strains were allowed to lay eggs onto plates at 16°C, grown to L4 stage, and shifted to the restrictive temperature 25°C for 24 hours.

## Supporting information

S1 Fig Radial locomotion assays were used to measure motor function following temperature-based inactivation of HSP-90. Animals were shifted to the restrictive temperature at L4 stage and assessed after 48 hours of HSP-90 inactivation. Animals expressing **A.** TDP-43^(M337V)^;*hsp-90(p673)*, **B.** TDP-43^(A315T)^;*hsp-90(p673)*, or **C.** TDP-43^(G290A)^;*hsp-90(p673)* show an improvement in their motility compared to controls without the *hsp-90* mutation. Error bars represent Mean with 95% confidence interval (CI): N = 3 independent experimental replicates; total n>100. Statistical significance as determined using Student’s t-test. (**** p<0.0001; **p<0.01). **D.** *hsp-90(p673)* does not suppress TDP-43 neuronal dysfunction without a shift to the restrictive temperature to inactivate HSP-90. Animals expressing fALS TDP-43^(M337V)^ alone or in combination with the *hsp-90(p673)* mutation were grown at 16°C. Radial locomotion assays were used to measure motor function. Error bars represent Mean with 95% CI: N = 3; n>100. Statistical significance as determined using Student’s t-test. (ns = not significant). **E.** 17-AAG treatment does not further improve TDP-43^(M337V)^;*hsp-90(p673)* motility. Animals expressing fALS TDP-43^(M337V)^;*hsp-90(p673)* mutation were grown at 16°C in the presence of 17-AAG to L4 stage, before a temperature shift to 25°C for 24 hours. Radial locomotion assays were used to measure motor function. Error bars represent Mean with 95% CI: N = 3; n>90. Statistical significance as determined using Student’s t-test. (ns = not significant).
(TIF)

S2 Fig Hsp90 mutation does not impact TDP-43 protein levels at the permissive temperature, 16°C. **A.** Representative immunoblots for total and phosphorylated TDP-43 in *C*. *elegans* populations maintained at 16°C**. B-C.** Mutant *hsp-90* does not decrease the levels of total and phosphorylated species of TDP-43 (pTDP-43). Error bars represent SEM: N = 4. Statistical significance as determined using unpaired t-test. (ns = not signficant) **D.** The ratio of phosphorylated TDP-43 to total TDP-43 (pTDP-43/ total TDP-43) is unchanged in TDP43^(M337V)^ versus TDP43^(M337V)^;*hsp-90(p673)*. **E.** Quantitative reverse-transcription PCR (qRT-PCR) testing expression of the TDP-43 transgene from animals grown at the permissive temperature, 16°C. TDP-43 signal is normalized to expression of an internal control gene, *rpl-32*, and plotted as arbitrary units (a.u.). **F.** qRT-PCR testing expression of the TDP-43 transgene from animals grown at the restrictive temperature, 25°C. TDP-43 signal is normalized to expression of an internal control gene, *rpl-32*, and plotted as arbitrary units. ns = not significant.
(TIF)

S3 Fig Heat shock does not protect against TDP-43 neurotoxicity. TDP-43^(M337V)^ were exposed to a 34°C heat shock at L4 stage, and allowed to recover for 24 hours at 20°C. Motility was tested using radial locomotion assay. Error bars represent SEM: N = 3; n = 90–101. Statistical significance determined by Student’s t-test. (**** p<0.0001).
(TIF)

S1 Table qRT-PCR primers. (TIF)
