# Supplementary figures and images for: Decreased Hsp90 activity protects against TDP-43 neurotoxicity in a C. elegans model of amyotrophic lateral sclerosis
Source: PLoS Genet. 2024 Dec 26;20(12):e1011518. doi: 10.1371/journal.pgen.1011518 (PMC11709271; doi:10.1371/journal.pgen.1011518)

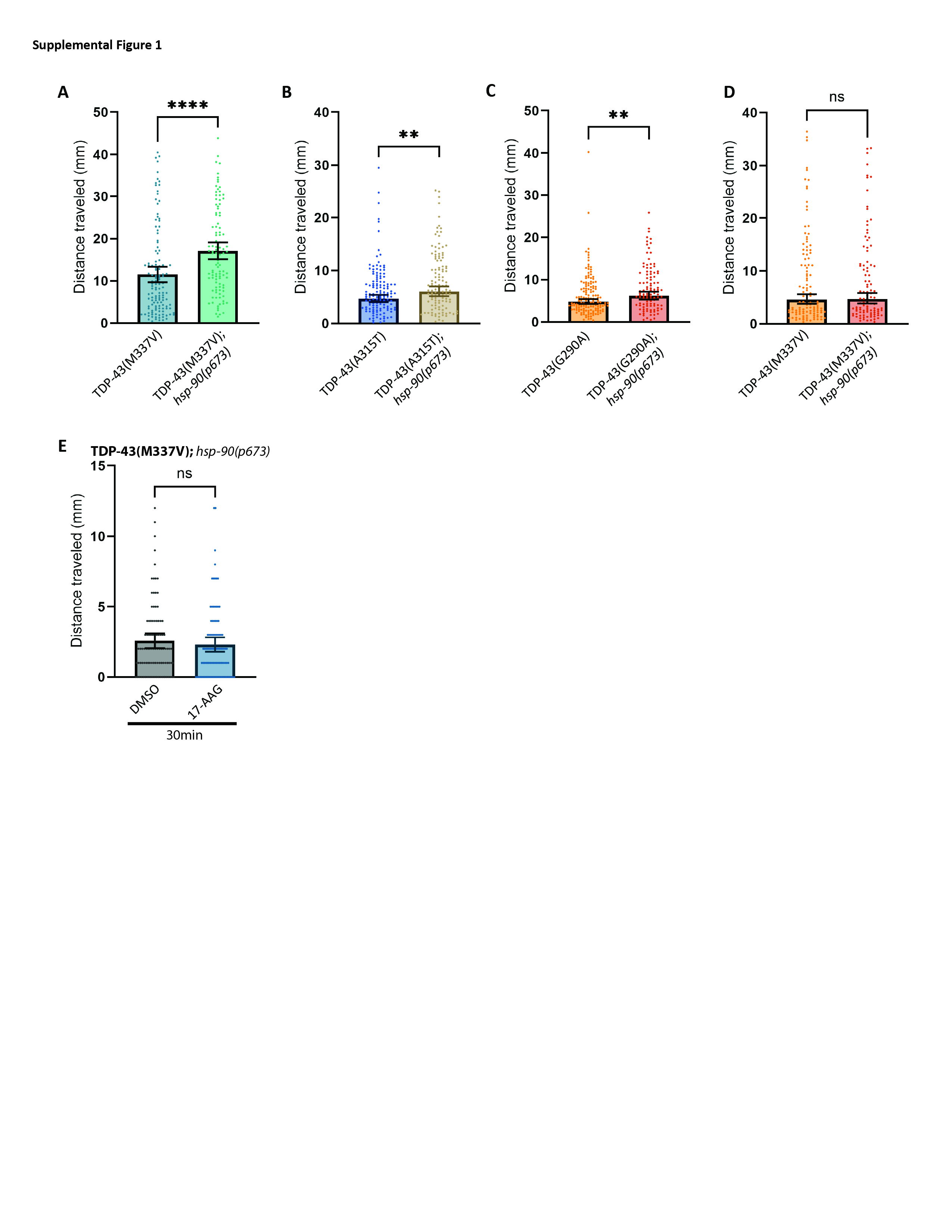

Supplement: S1 Fig — Animals were shifted to the restrictive temperature at L4 stage and assessed after 48 hours of HSP-90 inactivation. Animals expressing A. TDP-43(M337V);hsp-90(p673), B. TDP-43(A315T);hsp-90(p673), or C. TDP-43(G290A);hsp-90(p673) show an improvement in their motility compared to controls without the hsp-90 mutation. Error bars represent Mean with 95% confidence interval (CI): N = 3 independent experimental replicates; total n>100. Statistical significance as determined using Student’s t-test. (**** p<0.0001; **p<0.01). D. hsp-90(p673) does not suppress TDP-43 neuronal dysfunction without a shift to the restrictive temperature to inactivate HSP-90. Animals expressing fALS TDP-43(M337V) alone or in combination with the hsp-90(p673) mutation were grown at 16°C. Radial locomotion assays were used to measure motor function. Error bars represent Mean with 95% CI: N = 3; n>100. Statistical significance as determined using Student’s t-test. (ns = not significant). E. 17-AAG treatment does not further improve TDP-43(M337V);hsp-90(p673) motility. Animals expressing fALS TDP-43(M337V);hsp-90(p673) mutation were grown at 16°C in the presence of 17-AAG to L4 stage, before a temperature shift to 25°C for 24 hours. Radial locomotion assays were used to measure motor function. Error bars represent Mean with 95% CI: N = 3; n>90. Statistical significance as determined using Student’s t-test. (ns = not significant). (TIF) [file pgen.1011518.s001.tif]

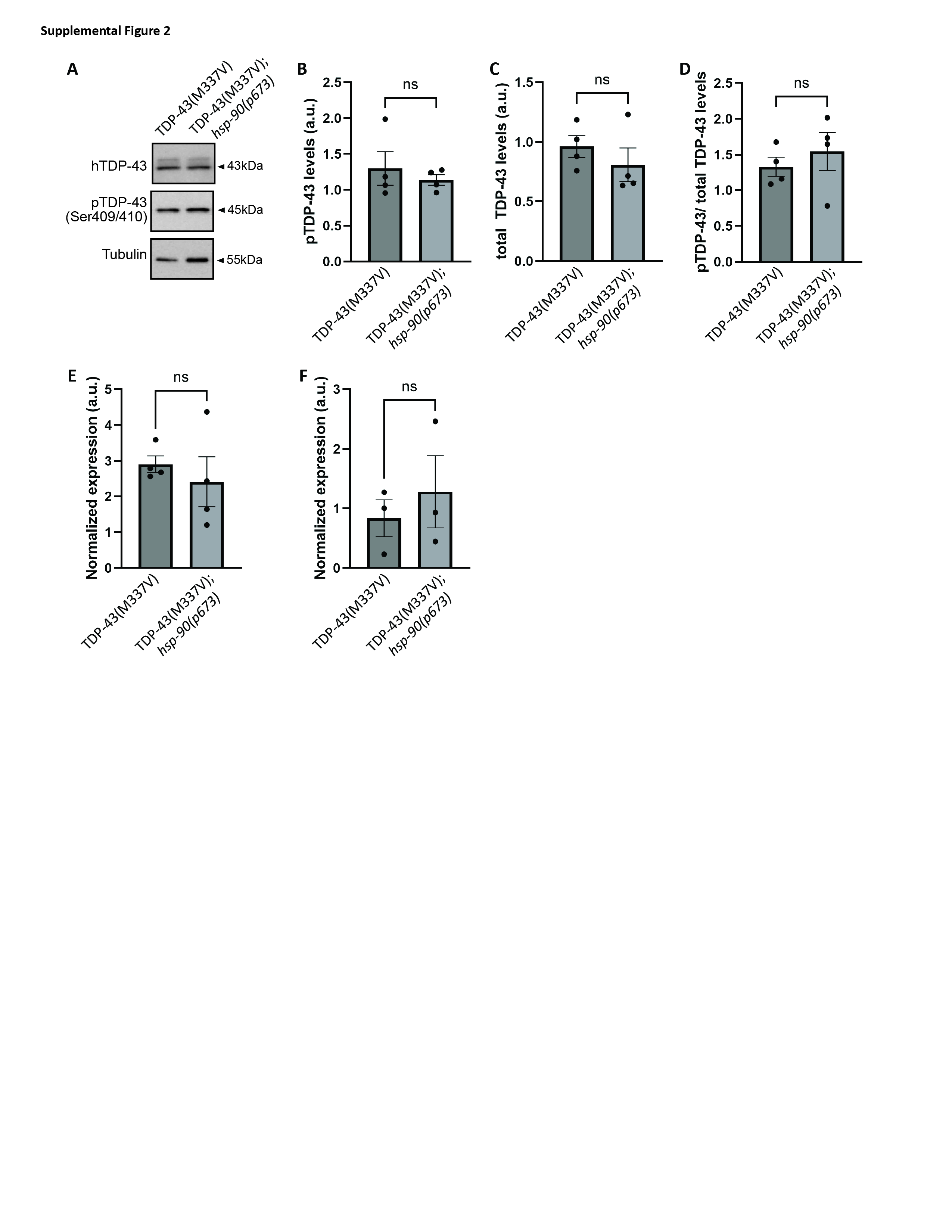

Supplement: S2 Fig — A. Representative immunoblots for total and phosphorylated TDP-43 in C. elegans populations maintained at 16°C. B-C. Mutant hsp-90 does not decrease the levels of total and phosphorylated species of TDP-43 (pTDP-43). Error bars represent SEM: N = 4. Statistical significance as determined using unpaired t-test. (ns = not signficant) D. The ratio of phosphorylated TDP-43 to total TDP-43 (pTDP-43/ total TDP-43) is unchanged in TDP43(M337V) versus TDP43(M337V);hsp-90(p673). E. Quantitative reverse-transcription PCR (qRT-PCR) testing expression of the TDP-43 transgene from animals grown at the permissive temperature, 16°C. TDP-43 signal is normalized to expression of an internal control gene, rpl-32, and plotted as arbitrary units (a.u.). F. qRT-PCR testing expression of the TDP-43 transgene from animals grown at the restrictive temperature, 25°C. TDP-43 signal is normalized to expression of an internal control gene, rpl-32, and plotted as arbitrary units. ns = not significant. (TIF) [file pgen.1011518.s002.tif]

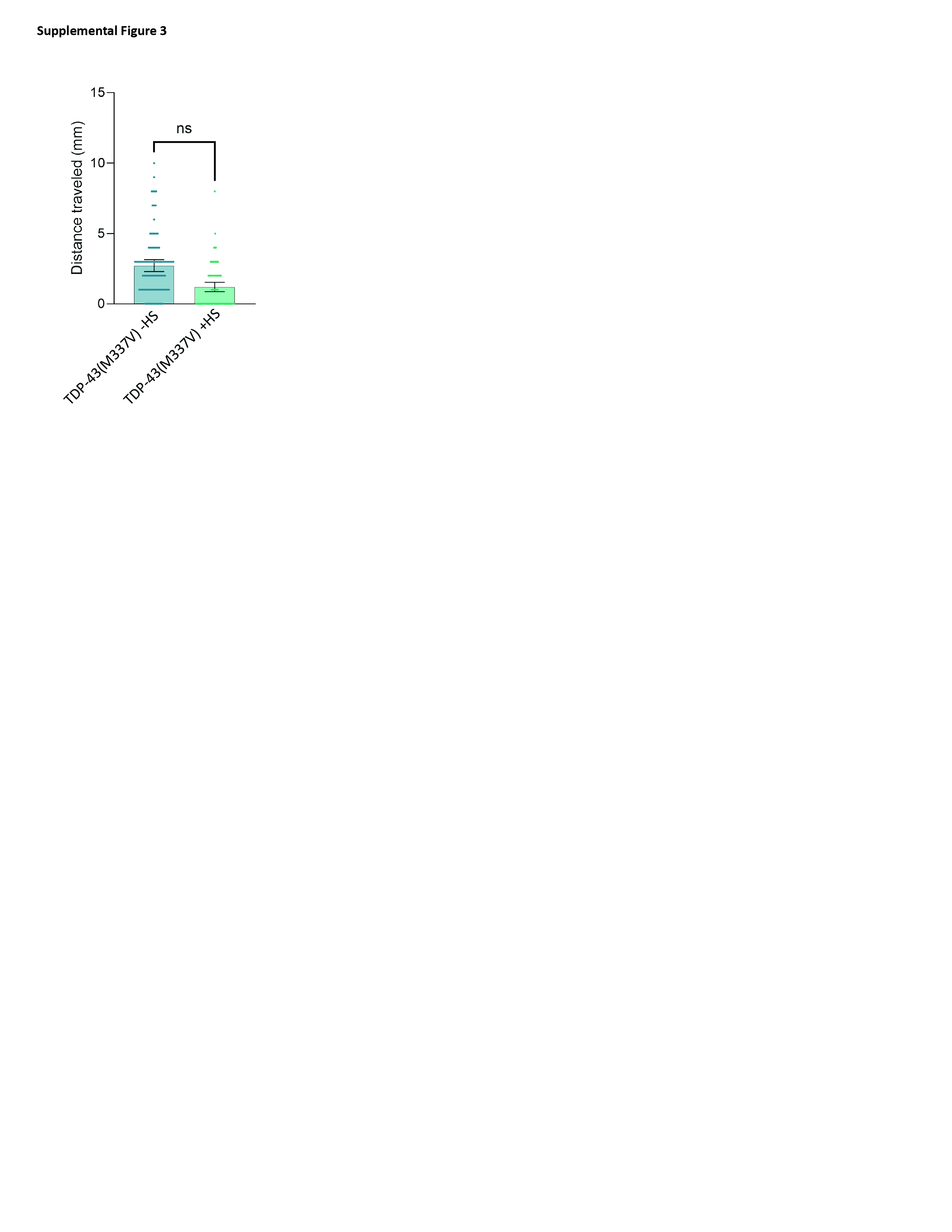

Supplement: S3 Fig — TDP-43(M337V) were exposed to a 34°C heat shock at L4 stage, and allowed to recover for 24 hours at 20°C. Motility was tested using radial locomotion assay. Error bars represent SEM: N = 3; n = 90–101. Statistical significance determined by Student’s t-test. (**** p<0.0001). (TIF) [file pgen.1011518.s003.tif]

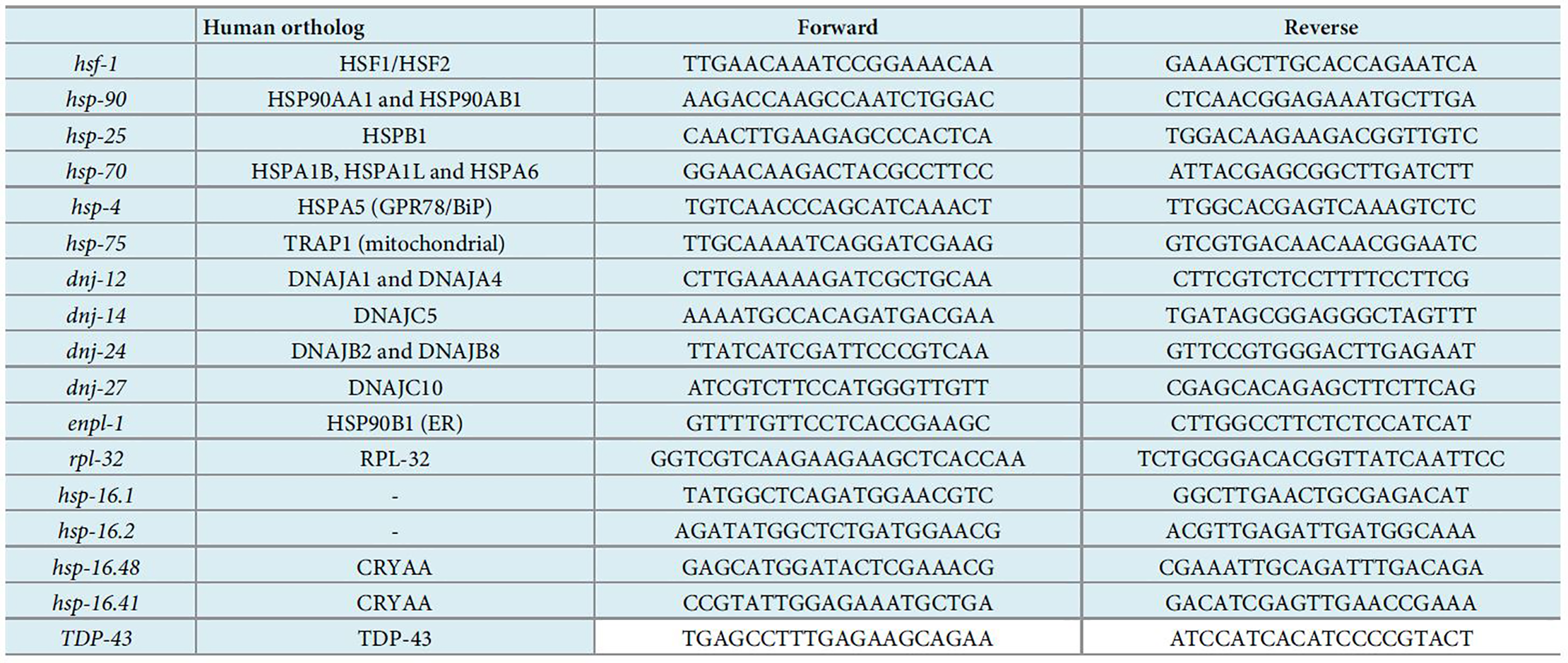

Supplement: S1 Table — (TIF) [file pgen.1011518.s004.tif]
